# Impact of Diagnosis of Urologic Cancer on Male Sexuality and Patients’ Perceptions of Health Professional's Approach

**DOI:** 10.1590/S1677-5538.IBJU.2025.9910

**Published:** 2025-03-25

**Authors:** Rodrigo Barros, Angelo Maurílio Fosse, Alex Schul, Gabriel Mangas, Caio Henrique da Silva Teixeira, Tiago Ninis, João Paulo Martins Carvalho, Luciano A. Favorito

**Affiliations:** 1 Universitário Antônio Pedro Serviço de Urologia do Hospital Niterói RJ Brasil Serviço de Urologia do Hospital Universitário Antônio Pedro - Universidade Federal Fluminense - UFF, Niterói, RJ, Brasil; 2 Serviço de Urologia do Hospital Federal Cardoso Fontes Rio de Janeiro RJ Brasil Serviço de Urologia do Hospital Federal Cardoso Fontes, Rio de Janeiro, RJ, Brasil; 3 Universidade do Estado do Rio de Janeiro Unidade de Pesquisa Urogenital Rio de Janeiro RJ Brasil Unidade de Pesquisa Urogenital - Universidade do Estado do Rio de Janeiro - UERJ, Rio de Janeiro, RJ, Brasil

**Keywords:** Urologic Neoplasms, Sexual Behavior, Quality of Life

## Abstract

**Objective:**

To evaluate the impact of a urological neoplasm diagnosis on male sexuality and examine patient perceptions of healthcare professionals’ engagement with sexuality at diagnosis.

**Materials and Methods:**

This study included adult men diagnosed with urological neoplasms across two urology cancer centers from October 2022 to April 2024. Exclusions applied to those with prior cancer treatment, inactive sexual lives, or histories of anxiety/depression treatment. Data were retrospectively gathered from medical records and individual interviews using a semi-structured questionnaire on male sexuality, relationship dynamics, and perceptions of physicians’ approach to sexuality. Analysis was conducted in RStudio using chi-square tests.

**Results:**

A cohort of 211 patients were included in this study, with an age range of 22 to 85 years (mean = 65.4). Among these patients, the diagnoses of urogenital cancer included cases of prostate (79.1%), bladder (9.9%), kidney (4.2%), testicular (3.7%), and penile (2.8%) neoplasms. Sexual activity was considered important by 90.5%, with 75.8% reporting decreased frequency post-diagnosis (99.5% were heterosexual and 72.5% were married). Anxiety or depression was reported by 46.9%, while 72% developed specific fears, primarily concerning erectile function and partner satisfaction. Only 23.2% of physicians addressed sexuality at diagnosis, with patient satisfaction significantly higher when these discussions occurred. Satisfaction levels were notably correlated with tumor site, with bladder cancer patients reporting higher satisfaction compared to those with prostate or penile cancer.

**Conclusions:**

Sexual issues extend beyond genital urological cancers, affecting patients with kidney and bladder cancers, with impacts often beginning at diagnosis. Early discussions on sexual health, combined with empathetic support, sexual education, and multidisciplinary care, are essential for the well-being of patients and their partners.

## INTRODUCTION

Sexual health is an important component of the quality of life, and there is an association between cancer and changes in sexual behavior resulting from the impact of the disease and its treatment (1). These changes can lead to significant distress, which at times can be the most difficult aspect of life following cancer.

Urological neoplasms often affect male sexuality, especially due to the physical consequences of their various therapeutic approaches, particularly in cases of prostate, testicle, and penile tumors. Indeed, the diagnosis of cancer can profoundly affect the emotional well-being of oncology patients, even in cases involving non-reproductive neoplasms, such as bladder and kidney cancers (2). Patients with other types of tumors that do not directly affect the sexual or reproductive functions, such as head and neck, lung and lymphatic cancers, can also experience sexual dysfunction and alterations to sexual self-esteem (3-6).

Education of the patient before treatment about sexual dysfunction related to the cancer can decrease the negative impact on sexual function after treatment. Nevertheless, most doctors do not address issues related to sexuality in the clinical context, instead focusing on treatment results, control of nonsexual adverse effects and patient survival (1). Previous research has reported mismatched expectations and unmet needs in relation to communication about sexuality between healthcare professionals and cancer patients (7). Oncosexology is a multidisciplinary field practiced by physicians, nurses, psychologists, and other healthcare professionals, with the intention of addressing sexual issues in patients with cancer (8). Therefore, sexual education, psychosocial and supportive care modalities can help manage distress, improve sexual function and quality of life of patients and their partners (9, 10).

The published literature on the impact of a cancer diagnosis on sexual functioning is relatively scarce. The objective of this study was to assess the impact of a urological neoplasm diagnosis on the sexuality of adult men and evaluate the patients’ perceptions of the healthcare professionals’ approach regarding sexuality at the time of diagnosis.

## MATERIAL AND METHODS

This study was approved by the ethics committee for human experimentation of our university (IRB number: 705.032). Adult men diagnosed with urological neoplasms in two urology cancer centers between October 2022 and April 2024, were invited to participate. All patients were diagnosed through biopsy except those with kidney cancer and superficial bladder cancer, who were diagnosed through imaging tests, and then confirmed with histopathology of the surgical specimen. Patients with a history of previous cancer treatment or who did not have an active sexual life prior to diagnosis were excluded from the study. Patients with a history of treatment for anxiety or depression were also excluded. Retrospective collection of demographic and oncological data was carried out through interviews and analysis of medical records. After obtaining the written informed consent, we conducted individual interviews to allow the participants to feel more comfortable and give us more accurate answers. The interviews were conducted using a semi-structured questionnaire prepared by a specialist in sexual medicine of the research team for the collection of objective clinical data. The questionnaire included changes in male sexuality after diagnosis of urological cancer, including sexual frequency, and development of fears or emotional symptoms (anxiety and depression). We also assessed modifications in relationship, behavior of partners or difficulty with couple communication. Finally, we evaluated the patient's perception of the doctor's approach regarding sexuality at the time of diagnosis.

Data were analyzed using the RStudio software. The association of the responses with each qualitative variable was measured using the chi-square test (95% confidence interval). A p-value <0.05 was considered statistically significant.

## RESULTS

A total of 211 patients were included in this study, with an age range of 22 to 85 years (mean = 65.4). Most were heterosexual (210 - 99.5%) and married (153 - 72.5%). Only 23 (10.9%) individuals had some level of college education. Among these patients, the diagnoses included prostate neoplasms in 167 cases (79.1%), bladder neoplasms in 21 cases (9.9%), kidney neoplasms in nine cases (4.2%), testicle neoplasms in eight cases (3.7%), and penis neoplasms in six cases (2.8%) Table-1.

**Table 1:** The table show the impact of the diagnosis of urologic cancer on male sexuality and patient's perception of the healthcare professional's approach.

TYPE OF CANCER	PROSTATE	BLADDER	KIDNEY	TESTICLE	PENIS
**N (%)**	164 (79.1%)	21 (9.9%)	9 (4.2%)	8 (3.7%)	6 (2.8%)
**Considered sexual activity important**	149 (90,1%)	17 (80.9%)	9 (100%)	7(87.5%)	6 (100%)
**Reported decreased sexual frequency**	127 (77,4%)	18 (85.7%)	6 (66.6%)	4 (50%)	6 (100%)
**Reported anxiety or depression**	77 (46,9%)	11 (52.3%)	3 (33.3%)	4 (50%)	2 (33.3%)
**Reported fear of losing their ability to maintain an erection**	83 (50,6%)	11 (52.3%)	3 (33.3%)	4 (50%)	3 (50%)
**Reported fearing the possibility of transmitting cancer**	48 (29,3%)	5 (23.8%)	0 (0%)	1(12.5%)	4 (66.6%)
**Reported communication difficulties with partner**	39 (23,8%)	16 (76.1%)	0 (0%)	0 (0%)	3 (50%)
**Doctors clearly discussed issues related to sexuality**	44 (26,8%)	2 (9.5%)	0 (0%)	4 (50%)	0 (0%)
**Expressed satisfaction with their doctors' approach**	71 (43,3%)	9 (42.8%)	1 (11.1%)	8 (100%)	1 (16.6%)

Sexual activity was considered important by 191 (90.5%) patients. The majority of them (160; 75.8%) reported decreased sexual frequency after diagnosis. In contrast, 49 patients (23.2%) maintained their pre-diagnosis frequency, while only two patients (0.9%) reported an increase. Furthermore, 143 (67.7%) respondents felt it was appropriate to continue sexual activity with their partners, while 68 (32.3%) did not. Ninety-nine (46.9%) patients experienced the onset of anxiety or depression after the diagnosis. Additionally, 152 patients (72%) developed various fears, with 108 (51.1%) expressing fear of losing their ability to maintain an erection, 55 (26%) fear of not satisfying the partner and 60 (28.4%) fearing the possibility of transmitting cancer to their partner. There was no statistically significant correlation between the type of cancer, aggressiveness or stage of the disease and the development of fear, anxiety or depression.

We found that 51 (24.2%) couples had communication difficulties. Eighty-four (39.8%) partners reported having increased closeness and intimacy, while 33 (15.6%) became more distant. Eight-nine (42.1%) did not change their behavior.

When we examined the relationship between patients and physicians, only 49 (23.2%) doctors clearly discussed issues related to sexuality at the time of diagnosis. Forty-one (19.4%) provided only superficial coverage of this topic, and 121 (57.3%) did not mention the subject at all. Interestingly, doctors did not address sexuality in 100% of kidney cancer cases and 75% of bladder cancer cases. In terms of patient satisfaction, 92 individuals (43.6%) expressed satisfaction with their doctors’ approach, while 119 (56.3%) were dissatisfied – Table-1.

There was a statistically significant correlation between patient satisfaction with medical care when it clearly addressed aspects of sexuality (p = 0.001). Moreover, there was a statistically significant association between satisfaction with the medical approach and the tumor site. Bladder tumor patients reported higher satisfaction, whereas prostate and penile cancer patients expressed greater dissatisfaction (p = 0.003).

## DISCUSSION

Receiving a cancer diagnosis is very trying for patients and their family. However, the impact of the cancer diagnosis on a patient's sexuality is often neglected by patients and doctors. The search for multiple medical opinions and/or the choice of different therapeutic options with the aim of curing cancer become a priority at this time. Nevertheless, sexual health support is essential to the well-being of patients with cancer and their partners (11). According to our sample, sexual activity was considered important by the majority of patients (90.5%).

The diagnosis of cancer can profoundly affect the emotional well-being of oncology patients, even in cases involving non-reproductive neoplasms. The fear of death and anticipation of the potential side effects of treatment, along with psychological and social factors, often deeply affect the quality of life of cancer patients. In particular, this diagnosis profoundly affects the sex life (12). In this study, the negative impact on male sexuality began at the time of diagnosis, before surgical approach or any type of treatment, probably due to anxiety, depression and fears related to cancer and its treatment.

The diagnosis of cancer has an impact on sexual relationships, and patients can reduce sexual activity and avoid sexual intimacy. Hawkins et al., evaluated changes in sexuality and intimacy after the diagnosis and treatment of cancer (13). Cessation or decreased frequency of sex and intimacy was reported by 79% of the men and 59% of the women. According to the authors, the reasons for changes in sexuality after cancer diagnosis were the impact of cancer treatments, exhaustion due to caring, and repositioning of the person with cancer as a patient, not a sexual partner. Moreover, sexual changes usually are associated with stress, fatigue and exhaustion, and most patients prioritize the treatment and survival than sexual life. In our sample, most patients (75.8%) decreased sexual frequency after diagnosis, while only two patients (0.9%) reported an increase. These two patients had prostate cancer and were afraid of developing erectile dysfunction after treatment.

The discovery of cancer can also trigger depression in up to 38% of patients and can be an important cause of sexual dysfunction, reduced sexual desire and difficulty of achieving erection. Lamentably, depressive symptoms can go undiagnosed and untreated (14). Psychological distress in cancer patients goes beyond emotional symptoms, such as anxiety and depression, and is associated with financial difficulties and spiritual problems. More than one-third of patients will suffer from moderate to severe distress during their cancer care (15). The prevalence of distress can differ according to the type of tumor, with patients with prostate, kidney and bladder cancers reporting 30%, 20% and 12% rates of clinically relevant distress respectively (15, 16). This last study reported that 99 (46.9%) men had anxiety or depression after the diagnosis of urological neoplasm. Therefore, psychosocial support and sexual education to address patients’ and partners should become an integral part of the multidisciplinary approach to treatment of cancer patients.

Sexual changes after cancer can impact couples’ relationships due to emotional distance or difficulty of communication between them (17). However, positive consequences of changes include increased closeness and intimacy. Unfortunately, the sexual quality of life of partners of patients with cancer is poorly understood and often neglected. Hence, clinicians should include the partner in the therapeutic process and referral for psychosexual treatment. In this study, many couples (24.2%) had communication difficulties. Eighty-four (39.8%) partners reported having increased closeness and intimacy, while 33 (15.6%) became more distant.

The lack of medical information, in addition to cultural and socioeconomic issues, can lead patients to develop different types of myths and beliefs. Older age can also contribute. Although younger patients typically have a fuller understanding of the disease through information from the Internet, most elderly people or those with lower academic qualifications and less income cannot distinguish between cancer and benign tumors (18). Likewise, in our study, the mean age was 65.4 years old and only a few individuals (10.9%) had some level of college education. More than a half of patients (55.4%) had only elementary schooling. This educational profile of patients is very common in public hospitals, like ours, because in our country people with higher education earn more and can better afford private health care. All told, 152 patients (72%) developed various fears, even the possibility of transmitting cancer to their partner. Moreover, sexual life should not be considered inappropriate in the context of cancer care, but rather be recognized as an aspect of couple relationships, which is associated with well-being (19). Physicians must take the time necessary to discuss sexual health with cancer patients fully. In our sample, 68 (32.3%) patients felt it was inappropriate to continue sexual activity with their partners after diagnosis of cancer.

The absence of discussion and information about sexuality at the diagnosis of cancer may negatively impact the patient's sexual quality of life. Although cancer diagnosis and treatment are critical issues, clinicians should discuss realistic expectations of the impact of cancer treatment on the patient's sexual function, the partner's sexual experience, and the couple's sexual relationship. Doctors cannot counsel a patients based on their beliefs rather than on reported data. According to a previous study, there is a difficulty of communication about sexuality and intimacy between patients and doctors (7). Even when sexuality is discussed, this is often not at a level that is satisfactory to most patients. In our study, only 23.2% of doctors clearly discussed issues related to sexuality at the time of diagnosis and 56.3% of individuals expressed discontent with their doctor's approach. These findings confirm previous research findings that these discussions are not conducted with most people with cancer, especially those outside the sphere of prostate cancer (20). Here, we also observed that doctors did not address sexuality in 100% of kidney cancer cases and 75% of bladder cancer cases.

Some obstacles can explain this lack of communication and difficulty in starting a conversation, such as priority in cancer treatment, embarrassment of the doctor to address the topic and silence of the patient on the subject (21). In addition, some professionals point out other barriers, such as lack of privacy, time and ability to discuss sexuality (22). Sometimes, physicians do not want to discuss sexuality in the presence of family or during caregiving for fear it would put the patient in an uncomfortable situation (7). It is worth noting that many patients do not express their concerns about their sex life, preferring the healthcare professionals to take the initiative by asking questions (23). Therefore, all members of the team that treats these patients must be proactive and prepared to address topics about sexuality. Information and sexual counseling can improve sexual function and reduce concern among patients and their partners (9, 10). Moreover, implementation of psychosocial interventions and participation of mental health care can have multiple benefits (24).

The present study has some limitations. First, we did not use a validated questionnaire, which can affect the accuracy and reliability of the data. Another limitation is the self-assessment declared through the questionnaire, since many patients may not feel comfortable disclosing certain information regarding their sexuality. Moreover, patients answered questions about changes in partner's behaviors from their own point of view. Additionally, we included patients diagnosed with different types of cancer in the same sample and thus subject to potential bias. However, the purpose of this study was achieved, and we identified negative effects of diagnosing urological neoplasms and the lack of sexual education of these patients. Furthermore, this study addressed the topic of major importance for the field of Oncosexology, highlighting the importance of adopting a multidisciplinary and centered approach to patients and their partners throughout the disease journey.

## CONCLUSION

Sexual problems are not limited to patients with genital urological cancer. Instead, they can also affect individuals with kidney and bladder cancer. The negative impact on male sexuality begins at the time of diagnosis, probably due to anxiety, depression and fears related to cancer and its treatment. Therefore, the treatment team should not ignore important quality of life issues and initiate discussion on sexual functioning soon following the initial diagnosis. Moreover, empathetic support, sexual education and multidisciplinary care are extremely important for these patients and their partners.
